# Data-driven Atlas of *Acinetobacter baumannii* vaccine research: a multi-database bibliometric and visualization analysis

**DOI:** 10.3389/fcimb.2026.1922186

**Published:** 2026-09-11

**Authors:** Shan Li, Xinyu Ju, Xingyu Zhu, Fan Fei, Xiaoqin Jin, Xingran Du, Yuting Shi

**Affiliations:** 1 Department of Respiratory and Critical Care Medicine, The Affiliated Jiangning Hospital of Nanjing Medical University, Nanjing, China; 2 Jiangning Clinical Medical College, Kangda College of Nanjing Medical University, Nanjing, China; 3 Department of Biochemistry and Molecular Biology, School of Laboratory Medicine, Bengbu Medical University, Bengbu, Anhui, China; 4 Department of Laboratory Medicine, The First Hospital of China Medical University, Shenyang, China

**Keywords:** *Acinetobacter baumannii*, antimicrobial resistance, bibliometric analysis, reverse vaccinology, vaccine development

## Abstract

**Introduction:**

*Acinetobacter baumannii* is a major multidrug-resistant pathogen for which effective preventive strategies are urgently needed. This study aimed to characterize the global research landscape, knowledge structure, and emerging trends in *Acinetobacter baumannii* vaccine research.

**Methods:**

Publications from 2011 to 2025 were retrieved from the Web of Science Core Collection and Scopus. After integration, deduplication, and independent screening, 243 publications were included. Bibliometric and visualization analyses were performed using bibliometrix, CiteSpace, and VOSviewer.

**Results:**

Research output showed an overall nonlinear increase, with an annual growth rate of 18.68%. Iran, China, and the United States were the leading contributors, whereas international collaboration accounted for 19.75% of publications. Keyword analysis identified seven major thematic clusters centered on outer membrane vesicles, bacterial antigens, antimicrobial resistance, computational approaches, and immunization strategies. Temporal analyses showed increasing attention to bioinformatics, reverse vaccinology, peptide/epitope-based design, and virulence-associated targets. Citation analysis further highlighted OMV/OMC-based vaccination and several defined antigens as important components of the field’s intellectual foundation.

**Discussion:**

Overall, *Acinetobacter baumannii* vaccine research has progressively diversified toward increasingly defined, multicomponent, and computationally guided strategies. However, antigenic heterogeneity, limited cross-strain validation, and variability in preclinical evaluation remain major translational challenges. Future efforts should emphasize conserved antigen prioritization, standardized validation, and broader international collaboration to advance promising candidates toward clinical application.

## 1 Introduction

*Acinetobacter baumannii* is a clinically important opportunistic pathogen responsible for healthcare-associated infections, including ventilator-associated pneumonia and bloodstream infections (Cisneros and Rodriguez-Bano, 2002; Alshehri et al., 2026). Owing to its remarkable antimicrobial resistance, environmental persistence, and rapid dissemination, it has become a major global public health threat (Lee et al., 2017; Hernandez-Bird et al., 2026).

In recent years, the widespread use of antimicrobials and ongoing evolution of resistance mechanisms have driven a marked rise in multidrug-resistant (MDR) and extensively drug-resistant (XDR) strains. At the same time, the efficacy of conventional therapies, including polymyxins and tigecycline, has steadily declined, rendering antibiotic-based treatment increasingly inadequate (Marino et al., 2024). As a result, preventive strategies such as vaccination have gained growing attention due to their ability to induce pathogen-specific immunity while reducing antibiotic dependence (Ma and Chen, 2021; Bjanes et al., 2023). Vaccine development efforts have explored multiple platforms, including inactivated whole-cell, subunit, DNA, outer membrane vesicle (OMV)-based, and multi-epitope vaccines. However, extensive antigenic heterogeneity, combined with complex virulence and immune evasion mechanisms, has limited broad protective efficacy and hindered clinical translation (Chen, 2020; Dey et al., 2023). The field has recently shifted from empirically driven approaches to data-driven vaccine discovery. The integration of reverse vaccinology, immunoinformatics, and artificial intelligence (AI) has significantly improved the identification of conserved, immunogenic antigens and accelerated the development of multi-epitope and precision vaccine strategies (Shahid et al., 2019; Liu et al., 2023; Jeffreys et al., 2024). Despite these advances, a systematic understanding of the global research landscape, knowledge structure, and thematic evolution in *Acinetobacter baumannii* vaccine research remains limited.

Bibliometric analysis offers a quantitative framework to map research development, identify emerging frontiers, and reveal patterns of international collaboration based on large-scale scientific data. Using integrated datasets from the Web of Science Core Collection (WoSCC) and Scopus, this study systematically examined the global landscape of *Acinetobacter baumannii* vaccine research. It focused on publication trends, collaboration networks, core themes, and emerging hotspots to provide a comprehensive overview of the field and inform future research and clinical translation.

## 2 Materials and methods

### 2.1 Data sources and data processing

Bibliometric data were retrieved from the Web of Science Core Collection (WoSCC) and Scopus databases to maximize data comprehensiveness and enhance the robustness of the findings. The search period spanned from 1 January 2011 to 31 December 2025, and only English-language publications classified as Article or Review were included. A Boolean search strategy was applied to identify publications related to *Acinetobacter baumannii* vaccines. The WoSCC search strategy was as follows: TS=(“*Acinetobacter baumannii*” OR “*A. baumannii*” OR “Bacterium anitratum”) AND TS=(“vaccin” OR “immunogenic”). The Scopus search strategy was: TITLE-ABS-KEY((“*Acinetobacter baumannii*” OR “*A. baumannii*” OR “Bacterium anitratum”) AND (“vaccin” OR “immunogenic”)) (**Supplementary Table 1**). We selected 2011 as the starting year because *Acinetobacter baumannii* vaccine research began to enter a more sustained and systematic phase around this period, with increasing attention to candidate antigen identification, immunogenicity evaluation, and protective efficacy assessment.

The initial search retrieved 399 records from WoSCC and 751 records from Scopus. The discrepancy in retrieval yields between the two databases can be attributed to several factors. First, WoSCC employs the TS (Topic) field, which searches titles, abstracts, and author keywords, whereas Scopus uses the TITLE-ABS-KEY field. Although these fields are conceptually comparable, Scopus provides broader coverage of journals, conference proceedings, and book chapters, with a larger indexed journal portfolio than WoSCC. Second, differences in document-type classification criteria and metadata annotation standards between the two databases may result in inconsistent categorization of identical records, thereby affecting the number of retained publications after screening. Third, Scopus generally provides more extensive coverage of regional journals and English-language publications from non-English-speaking countries, many of which may not be indexed in the WoSCC core collection.

Accordingly, after applying consistent inclusion criteria regarding language, document type, and publication period, the final numbers of eligible records differed between WoSCC (351 publications) and Scopus (626 publications). This discrepancy likely reflects inherent differences in database coverage characteristics rather than inconsistencies in the literature retrieval process.

### 2.2 Literature screening

The records retrieved from the two databases were merged and deduplicated using the bibliometrix package in R and customized Python scripts. Duplicate records were identified based on bibliographic metadata, including titles, authors, and publication years. After removing 282 duplicate records, a total of 695 unique publications were obtained.

**Figure 1:**
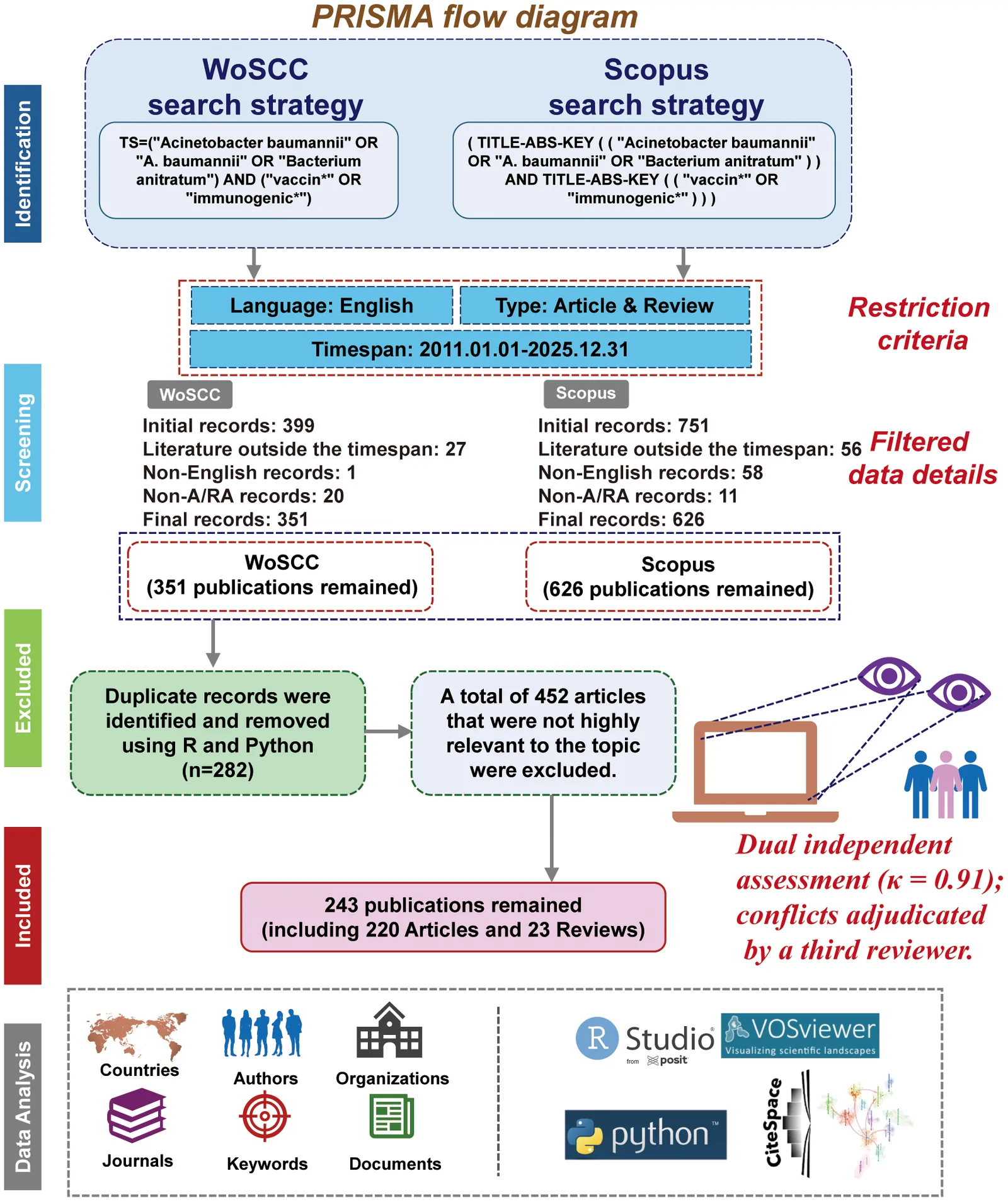
Literature screening and inclusion workflow. This flowchart illustrates the process of literature retrieval, deduplication, manual screening, and final inclusion for analysis. After applying the predefined restrictions on language, document type, and publication period, 351 records from WoSCC and 626 records from Scopus were retained. After merging the search results, 282 duplicate records were removed using R and Python, yielding 695 unique records. Two researchers then independently assessed the relevance of each record to the study topic, with a third researcher adjudicating any discrepancies; inter-rater agreement was good (κ = 0.91). Following manual screening, 452 records deemed insufficiently relevant to the study topic were excluded, resulting in a final set of 243 publications included in the bibliometric analysis, comprising 220 original research articles and 23 reviews.

Subsequently, two independent researchers manually screened the titles and abstracts of all 695 publications against predefined inclusion and exclusion criteria to assess their relevance to *Acinetobacter baumannii* vaccine research. Inter-rater agreement between the two reviewers was assessed using Cohen’s kappa coefficient (κ = 0.91, 95% CI: 0.89–0.93), indicating a high level of agreement. Any discrepancies were resolved through adjudication by a third researcher. Following the screening process, 452 publications deemed insufficiently relevant were excluded, leaving 243 publications, comprising 220 articles and 23 reviews, for subsequent bibliometric analyses (**Figure 1**). The complete bibliographic information of the included publications after screening has been provided in **Supplementary Table 2**.

The inclusion criteria were as follows: (i) studies directly investigating *Acinetobacter baumannii* vaccines, including vaccine design, antigen identification, immunogenicity evaluation, protective efficacy assessment, or related immune mechanisms; (ii) publications classified as original research articles or reviews; (iii) publications written in English; and (iv) publications published between 1 January 2011 and 31 December 2025.

The exclusion criteria were as follows: (i) studies without a direct focus on *Acinetobacter baumannii* vaccines, including publications that only mentioned terms such as “vaccine” or “immunogenic” in the introduction or discussion while primarily addressing other topics, such as antimicrobial resistance, epidemiology, or diagnostic methods; (ii) conference abstracts, editorials, comments, corrections, and other non-Article/Review publication types; (iii) publications with incomplete data; and (iv) duplicate publications or redundant reports.

To minimize potential bias in keyword co-occurrence analysis and thematic clustering caused by review articles that were not specifically focused on *Acinetobacter baumannii* vaccine development, additional eligibility criteria were applied to review articles during the title and abstract screening process. Specifically, only reviews whose primary content centered on *Acinetobacter baumannii* vaccine-associated antigens, immunization strategies, or preclinical/clinical advances were considered eligible. All 23 included reviews fulfilled these criteria.

### 2.3 Bibliometric analysis software and parameter settings

A suite of bibliometric tools was used to comprehensively characterize the research landscape of *Acinetobacter baumannii* vaccine studies. Descriptive analyses were performed using the bibliometrix package (v5.0) in R (v4.4.5), including annual publication output, citation metrics, distributions of countries, authors, and journals, as well as citation analyses. Network data were extracted for visualization, and thematic evolution bubble maps were generated to illustrate temporal shifts in research focus (Arruda et al., 2022).

Keyword co-occurrence clustering was further conducted using CiteSpace (v6.4.R1). The time-slicing interval was set to one year, and node selection followed a g-index (k = 25) with pruning parameters LRF = 2.5, L/N = 10, LBY = 5, and e = 1.0. Keyword and citation burst detection was performed with a minimum burst duration of one year, while other parameters (α = 2.0, γ = 0.65) were kept at default settings (Synnestvedt et al., 2005).

Author co-citation networks were constructed using VOSviewer (v1.6.20), with inclusion criteria of at least five publications and no citation threshold (citations ≥ 0) (Arruda et al., 2022). Outputs from these tools were further processed and visualized in Python (v3.14) using the pandas, matplotlib, seaborn, and networkx libraries.

Institutional productivity was assessed using full counting, which may inflate counts due to multiple affiliations per publication. In contrast, country-level analyses used fractional counting, where contributions were proportionally allocated based on unique publication identifiers, providing a more accurate estimate of national research output.

## 3 Results

### 3.1 Overall landscape and publication trends

**Figure 2:**
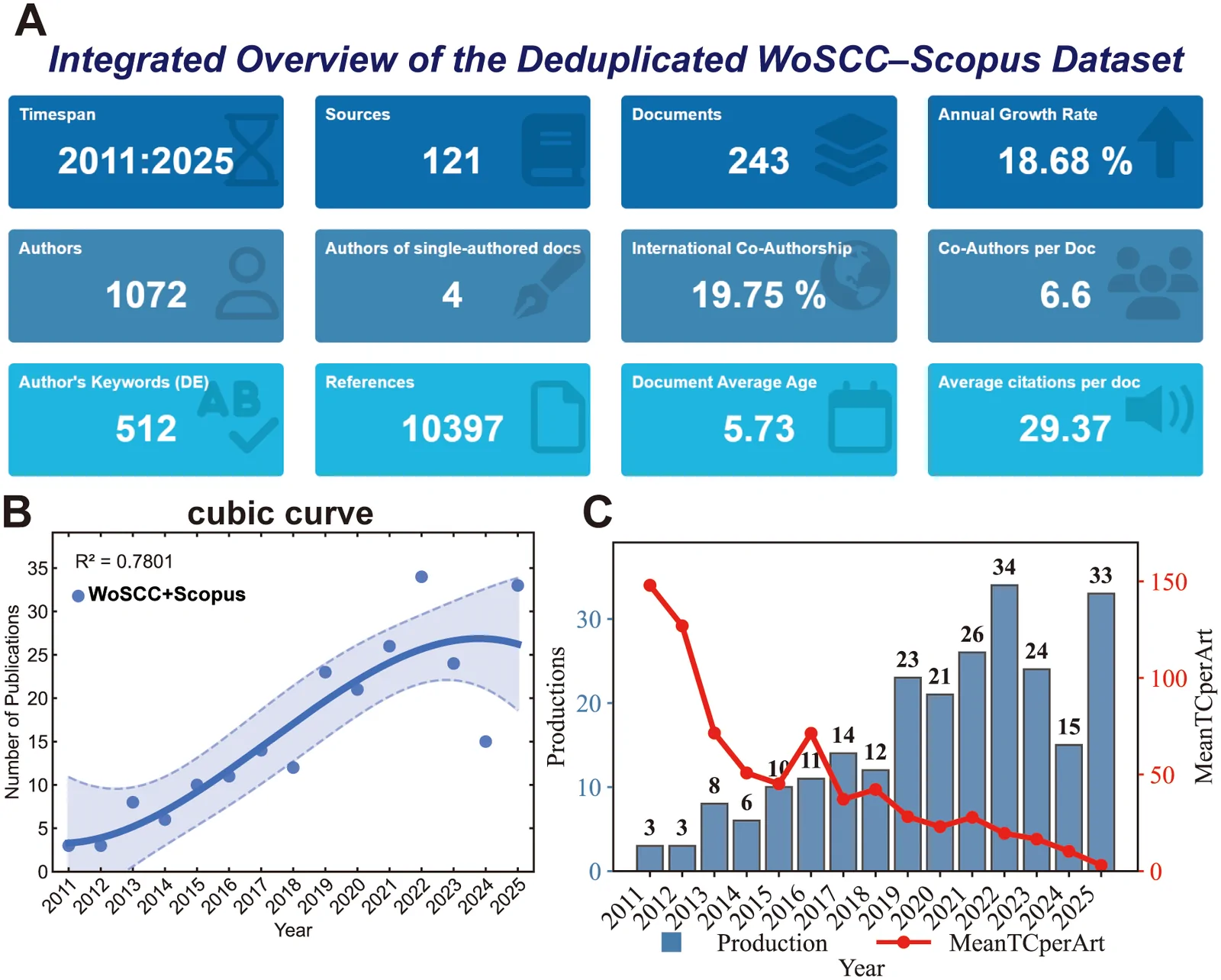
Bibliometric characteristics and temporal trends of research on *Acinetobacter baumannii* vaccines, 2011–2025. **(A)** Overview of the integrated, deduplicated WoSCC–Scopus dataset (243 documents, 2011–2025), including the number of sources (121), authors (1,072, of whom 4 were single-document authors), annual growth rate (18.68%), international co-authorship rate (19.75%), co-authors per document (6.6), author keywords (512), references (10,397), average document age (5.73 years), and average citations per document (29.37). **(B)** Annual publication trend from 2011 to 2025, fitted with a cubic curve (R² = 0.7801); the shaded area represents the 95% confidence interval, and dots indicate the actual number of publications each year. **(C)** Annual number of publications (bar chart, left y-axis) compared with the mean total citations per article (MeanTCperArt; red line, right y-axis) from 2011 to 2025; numbers above the bars indicate the annual publication count.

Based on the integrated and deduplicated dataset from the WoSCC and Scopus databases, a total of 243 publications on *Acinetobacter baumannii* vaccine research published between 2011 and 2025 were included, spanning 121 publication sources and involving 1,072 authors. Among these authors, four contributed to single-authored documents, while the average number of co-authors per document was 6.6. The international collaboration rate was 19.75%, indicating that approximately one-fifth of the publications involved international collaboration. The dataset contained 10,397 cited references and 512 author keywords. The average document age was 5.73 years, and the average number of citations per document was 29.37. The annual publication growth rate was 18.68% (**Figure 2A**).

Regarding publication dynamics (**Figure 2B**), the annual output of *Acinetobacter baumannii* vaccine-related publications exhibited an overall nonlinear upward trend from 2011 to 2025. A third-order polynomial regression model fitted to the annual publication output yielded an R² value of 0.7801. Annual output remained relatively low during 2011–2013, ranging from 3 to 8 publications. Thereafter, publication activity generally increased despite year-to-year fluctuations, exceeding 20 publications for the first time in 2019 (23 publications). Following a slight decrease to 21 publications in 2020, the annual output increased to 26 in 2021 and peaked at 34 in 2022. It subsequently declined to 24 in 2023 and 15 in 2024, before rebounding to 33 publications in 2025.

Analysis of annual publication output alongside the mean total citations per article (MeanTCperArt) showed that publications from 2011 and 2012, despite the small number of publications in each year (n = 3), had the highest MeanTCperArt values during the study period, at 148 and 127 citations per article, respectively (**Table 1**; **Figure 2C**). These publications also had the longest citable periods, corresponding to 16 and 15 citable years, respectively. Across subsequent publication years, MeanTCperArt exhibited an overall downward trend with some year-to-year fluctuations, reaching 3.00 citations per article in 2025. More recent publications had progressively shorter citable periods.

**Table 1:** Annual publication output and citation metrics, 2011–2025.

Year	MeanTCperArt	N	MeanTCperYear	CitableYears
2011	148.00	3	9.25	16
2012	127.00	3	8.47	15
2013	71.50	8	5.11	14
2014	50.83	6	3.91	13
2015	45.20	10	3.77	12
2016	71.36	11	6.49	11
2017	37.21	14	3.72	10
2018	42.25	12	4.69	9
2019	28.17	23	3.52	8
2020	23.00	21	3.29	7
2021	27.85	26	4.64	6
2022	19.56	34	3.91	5
2023	16.58	24	4.14	4
2024	10.27	15	3.42	3
2025	3.00	33	1.50	2

### 3.2 Core contributors, geographic distribution, and institutional and journal profiles

The 243 included publications were distributed across 121 journals or publication sources and involved 1,072 authors. Bibliometric analysis was performed to systematically identify the major contributors, leading countries, prominent journals, and key institutions shaping the landscape of Acinetobacter baumannii vaccine research.

**Figure 3:**
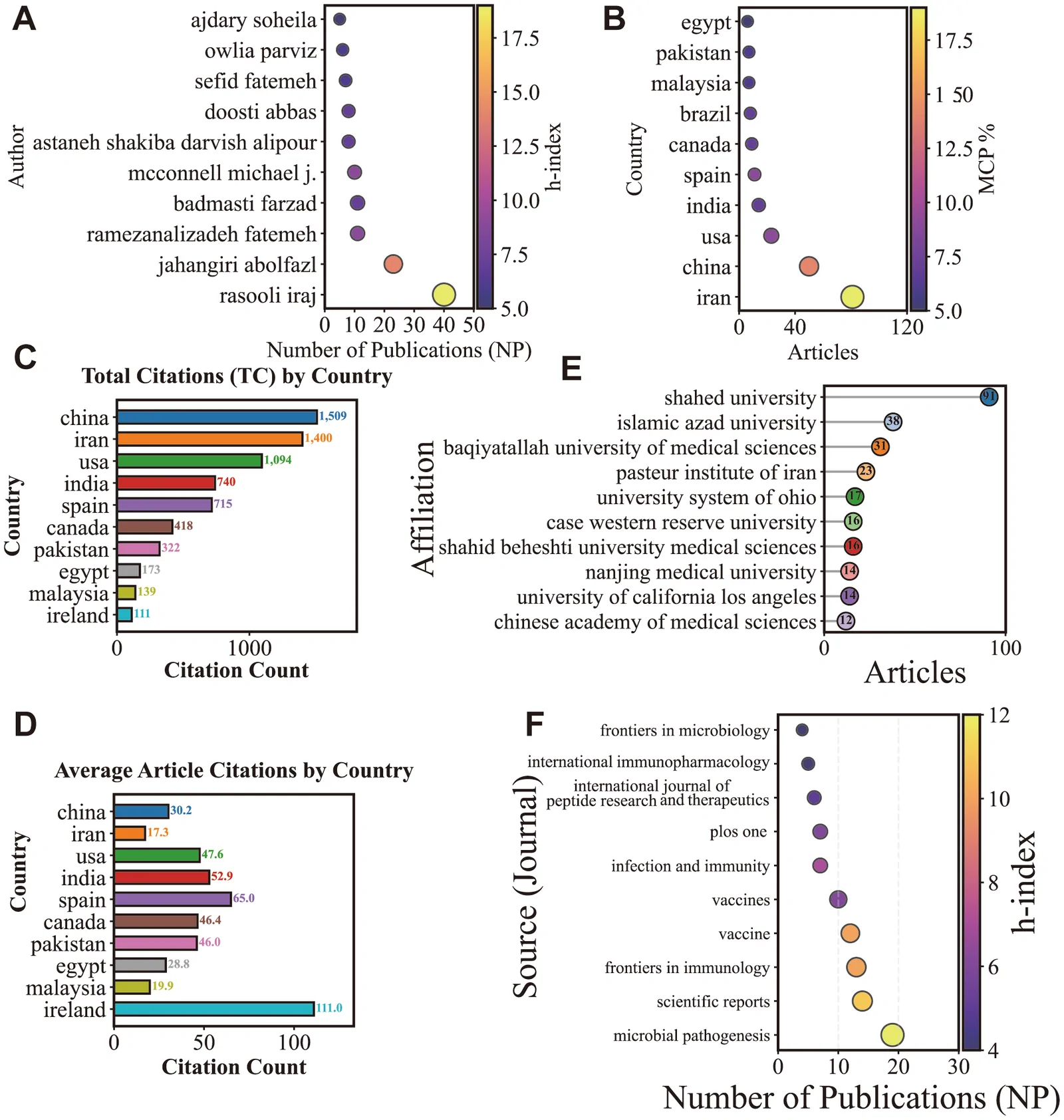
Core contributors, geographic distribution, citation impact, institutional output, and leading journals in research on *Acinetobacter baumannii* vaccines. **(A)** Top 10 most productive authors ranked by number of publications (NP), with bubble color indicating each author's h-index. **(B)** Top 10 countries ranked by number of publications, with bubble color indicating the international co-authorship rate (MCP%, the proportion of multiple-country publications). **(C)** Countries ranked by total citations (TC), with China (1,509) and Iran (1,400) ranking first and second, respectively. **(D)** Average citations per article by country. **(E)** Top 10 institutions ranked by number of publications, with the numbers within the bubbles indicating publication counts. **(F)** Top 10 source journals ranked by number of publications, with bubble color indicating journal h-index.

Among individual contributors, Rasooli Iraj had the highest number of publications, with 40 articles, followed by Jahangiri Abolfazl with 23. Rasooli Iraj also had the highest h-index of 19, g-index of 28, and total citation count of 880. In terms of citation count, McConnell Michael J. ranked second with 574 total citations based on 10 publications. Ramezanalizadeh Fatemeh had the highest m-index of 1.286 (**Figure 3A**; **Table 2**).

**Table 2:** Top ten authors ranked by bibliometric indicators.

Author	h_index	g_index	m_index	TC	NP	PY_start
rasooli iraj	19	28	1.188	880	40	2011
jahangiri abolfazl	14	20	1.167	436	23	2015
mcconnell michael j.	9	10	0.563	574	10	2011
ramezanalizadeh fatemeh	9	11	1.286	190	11	2020
astaneh shakiba darvish alipour	7	8	0.438	287	8	2011
badmasti farzad	7	11	0.583	187	11	2015
doosti abbas	7	8	0.7	147	8	2017
owlia parviz	6	6	0.6	208	6	2017
sefid fatemeh	6	7	0.5	140	7	2015
ajdary soheila	5	5	0.417	122	5	2015

At the country level, publication output was unevenly distributed (**Figure 3B**; **Table 3**). Iran contributed the largest number of publications, with 81 articles accounting for 33.3% of the total, followed by China with 50 articles (20.6%) and the United States with 23 (9.5%). Canada had the highest proportion of multiple-country publications (MCP%) (66.7%), followed by Egypt (50%), Malaysia, and Pakistan (both 42.9%). In contrast, Iran and China had lower MCP percentages of 7.4% and 10.0%, respectively, indicating that most publications from these two countries were single-country publications.

**Table 3:** Top ten contributing countries by publication output and international collaboration.

Country	Articles	Articles %	SCP	MCP	MCP %
iran	81	33.3	75	6	7.4
china	50	20.6	45	5	10
usa	23	9.5	15	8	34.8
india	14	5.8	13	1	7.1
spain	11	4.5	11	0	0
canada	9	3.7	3	6	66.7
brazil	8	3.3	8	0	0
malaysia	7	2.9	4	3	42.9
pakistan	7	2.9	4	3	42.9
egypt	6	2.5	3	3	50

Citation patterns varied across countries (**Figure 3C**). China had the highest total citation count, with 1,509 citations, followed by Iran with 1,400 and the United States with 1,094. Average citations per article showed a different distribution (**Figure 3D**). Ireland had the highest average citation count at 111.0 citations per article, followed by Spain at 65.0 and India at 52.9. Despite ranking among the leading countries in publication output and total citations, China and Iran had average citation counts of 30.2 and 17.3 citations per article, respectively. Canada and Pakistan recorded comparable averages of 46.4 and 46.0 citations per article, respectively.

For institutional analysis, publication counts were calculated using the full-counting method, in which each institution listed in a publication was counted once. In contrast, country-level analysis was conducted based on publication attribution, with each article assigned once to each corresponding country. Therefore, because individual publications may involve multiple institutions, the publication count of highly collaborative institutions may exceed the total number of publications attributed to their respective countries (e.g., Shahed University contributed 91 publications, exceeding the 81 publications attributed to Iran). This discrepancy reflects differences in counting methodologies rather than inconsistencies in the dataset.

Institutional analysis further demonstrated that Shahed University was the leading contributor, with 91 publications, followed by Islamic Azad University (38 publications) and Baqiyatallah University of Medical Sciences (31 publications) (**Figure 3E**). These findings indicate a strong institutional concentration of research activity within Iran. In addition, Pasteur Institute of Iran (23 publications), the University System of Ohio in the United States (17 publications), and Case Western Reserve University (16 publications) represented important contributors. Meanwhile, sustained contributions from Nanjing Medical University (14 publications) and the University of California, Los Angeles (14 publications) further illustrate the increasingly diverse international participation in this field.

Journal-level analysis showed that Microbial Pathogenesis had the highest publication output, with 19 articles and an h-index of 12, followed by Scientific Reports with 14 articles and an h-index of 11 and Frontiers in Immunology with 13 articles and an h-index of 10. Vaccine published 12 articles, with an h-index of 10 and a total citation count of 623 (**Figure 3F**; **Table 4**). The distribution of publication venues highlights the interdisciplinary nature of Acinetobacter baumannii vaccine research, with findings disseminated across microbiology, immunology, and vaccinology.

**Table 4:** Top ten journals ranked by bibliometric indicators.

Source	h_index	g_index	m_index	TC	NP	PY_start
microbial pathogenesis	12	19	0.75	506	19	2011
scientific reports	11	14	1	786	14	2016
frontiers in immunology	10	13	1.429	263	13	2020
vaccine	10	12	0.625	623	12	2011
infection and immunity	7	7	0.438	556	7	2011
plos one	6	7	0.4	520	7	2012
vaccines	6	10	1	217	10	2021
international journal of peptide research and therapeutics	5	6	0.625	125	6	2019
frontiers in microbiology	4	4	0.364	216	4	2016
international immunopharmacology	4	5	0.8	51	5	2022

### 3.3 Analysis of author, institutional, and journal productivity

**Figure 4:**
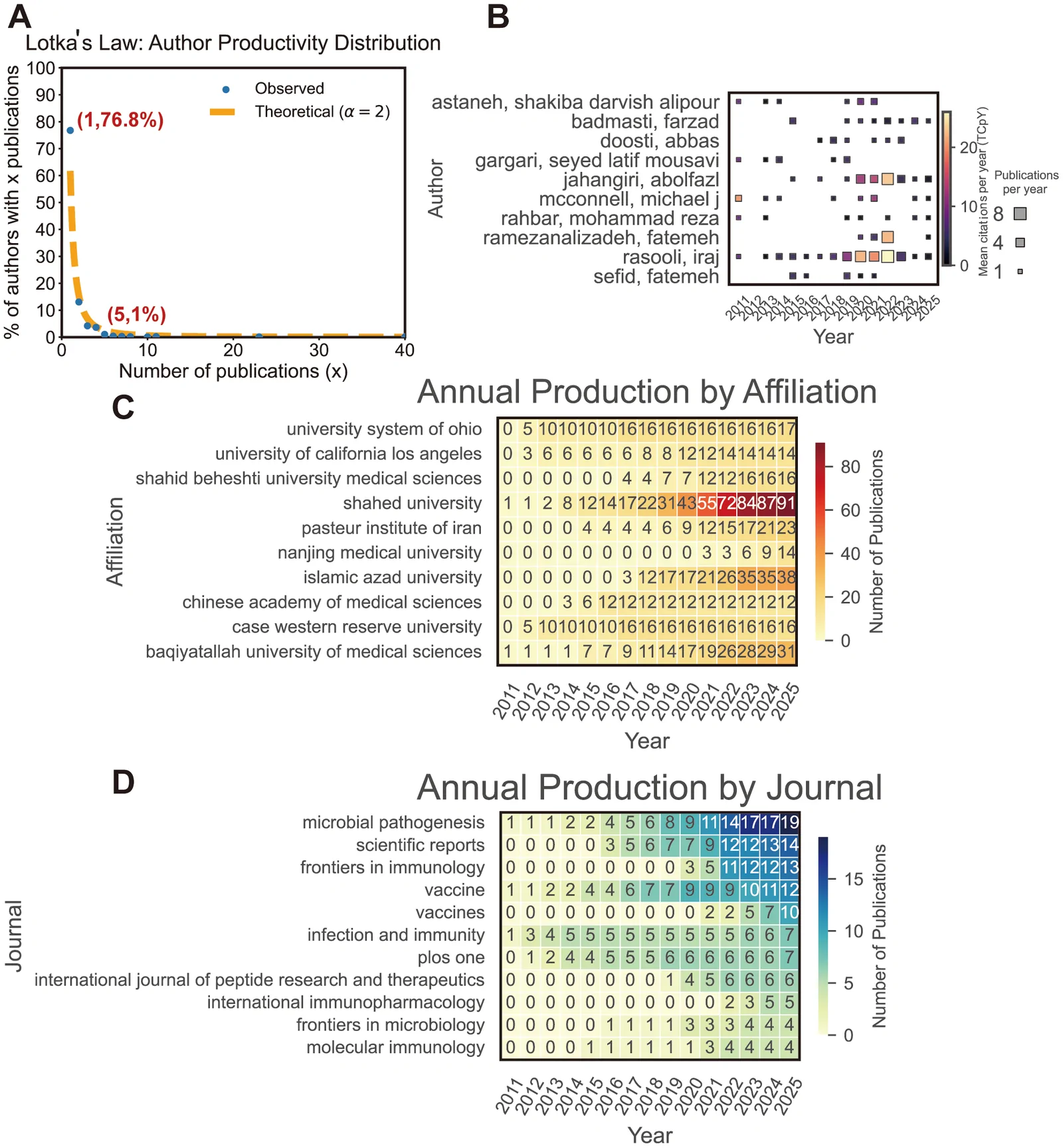
Distribution of author productivity and the temporal evolution of publication output among authors, institutions, and journals. **(A)** Lotka's Law analysis showing that 76.8% of authors published 1 paper, while 1.0% of authors published 5 papers. **(B)** Annual publication trends of the top 10 most productive authors. **(C)** Annual cumulative publication trends of the top 10 institutions (full counting method). **(D)** Annual cumulative publication trends of the top 10 journals.

Using the bibliometrix package in R, we characterized the distribution of author productivity and evaluated the longitudinal publication patterns of major authors, institutions, and journals in the field of *Acinetobacter baumannii* vaccine research. Lotka’s Law analysis revealed a highly skewed author productivity distribution (**Figure 4A**). Among the 1,072 identified authors, 76.8% contributed only one publication, whereas the proportion of authors with five or more publications was approximately 1%. Only a small number of researchers, including Rasooli Iraj and Jahangiri Abolfazl, contributed more than 10 publications, highlighting the concentration of research output among a limited group of highly productive investigators.

The temporal publication profiles of leading authors were further assessed based on annual publication frequency (Freq) and total citations per year (TCpY) (**Figure 4B**; **Supplementary Table 3**). Rasooli Iraj showed sustained publication activity over more than a decade, with publications recorded in 14 years between 2011 and 2025. His annual outputs during 2019–2022 were 4, 6, 5, and 8 publications, with corresponding TCpY values of 10.125, 21.857, 19.333, and 26.0, respectively. Both annual publication frequency and TCpY peaked in 2022. During 2023–2025, his publication counts decreased to 4, 1, and 2 articles, accompanied by TCpY values of 5.5, 1.333, and 1.5, respectively. Jahangiri Abolfazl showed increased productivity during 2020–2022, publishing 4, 3, and 7 articles annually, with corresponding TCpY values of 11.857, 13.667, and 23.0. From 2023 to 2025, his publication output declined to 3, 1, and 2 articles, with TCpY values of 4.0, 3.0, and 1.5, respectively. Ramezanalizadeh Fatemeh demonstrated concentrated research activity during 2020–2022 and 2025, with the highest annual output observed in 2022 (7 publications; TCpY = 22.0). In the years in which he published, McConnell Michael J. generally contributed one article, except in 2011 and 2021, when he published two articles. His 2011 publications achieved the highest citation impact across his publication history (TCpY = 20.063). Other contributors, including Astaneh Shakiba Darvish Alipour, Badmasti Farzad, Doosti Abbas, Gargari Seyed Latif Mousavi, Rahbar Mohammad Reza, and Sefid Fatemeh, typically published 1–2 articles annually, with TCpY values generally below 10. Their publication activity was comparatively sparse, particularly after 2022.

At the institutional level, publication productivity was assessed using the full-counting method (**Figure 4C**). Shahed University exhibited a marked increase in research output, from one publication in 2011 to a cumulative total of 91 publications by 2025. Islamic Azad University and Baqiyatallah University of Medical Sciences ranked next, with 38 and 31 publications, respectively. Other notable contributors included the University System of Ohio (17 publications), Case Western Reserve University (16 publications), and the University of California, Los Angeles (14 publications).

Journal-level analysis demonstrated that Microbial Pathogenesis was the most productive publication venue, increasing from one article in 2011 to 19 cumulative publications by 2025 (**Figure 4D**). Scientific Reports and Frontiers in Immunology began publishing relevant studies around 2016 and 2020, respectively, and accumulated 14 and 13 publications by 2025. Vaccine published 12 related articles, whereas Vaccines began contributing to this field around 2021 and reached 10 publications by 2025.

### 3.4 Collaboration networks among authors, countries, and institutions

Using the bibliometrix package in R, we visualized the collaboration networks among authors, countries, and institutions in *Acinetobacter baumannii* vaccine research. The betweenness centrality of each node was calculated to quantify its role as a bridge connecting different components within the collaboration networks.

**Figure 5:**
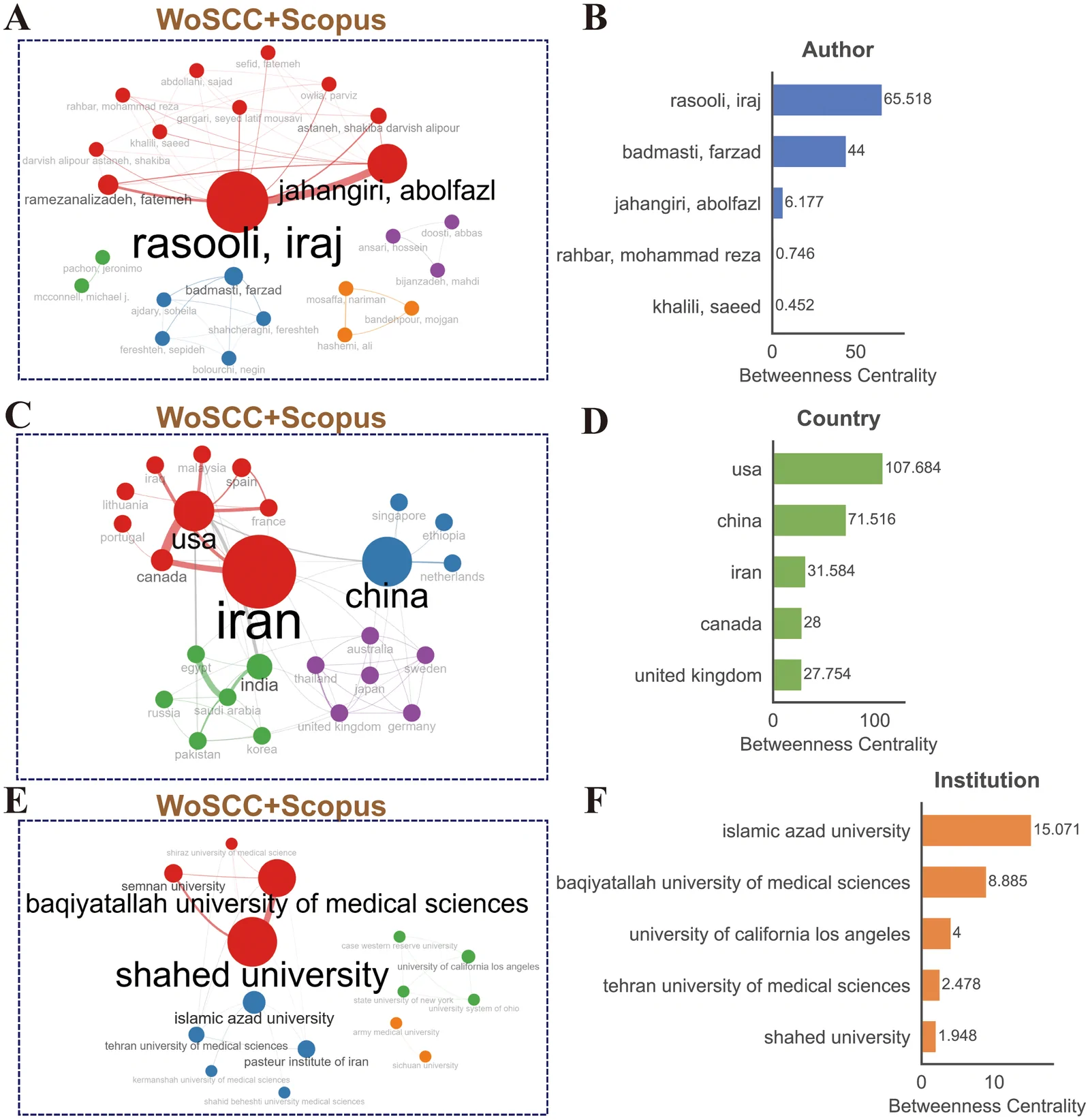
Collaboration networks and betweenness centrality analysis of authors, countries, and institutions based on the combined WoSCC and Scopus dataset. **(A)** Author collaboration network constructed from the combined WoSCC and Scopus dataset, in which Rasooli Iraj and Jahangiri Abolfazl occupy relatively prominent positions. **(B)** Top 5 authors ranked by betweenness centrality, with Rasooli Iraj ranking highest. **(C)** Country collaboration network constructed from the combined dataset, with Iran, the United States, and China serving as the main collaboration hubs. **(D)** Top 5 countries ranked by betweenness centrality, with the United States ranking first, followed by China and Iran. **(E)** Institutional collaboration network constructed from the combined dataset, in which Shahed University and Baqiyatallah University of Medical Sciences occupy relatively prominent positions. **(F)** Top 5 institutions ranked by betweenness centrality, with Islamic Azad University ranking highest, followed by Baqiyatallah University of Medical Sciences.

The author collaboration network (**Figure 5A**) revealed several distinct collaboration clusters. The largest cluster was centered around Rasooli Iraj and Jahangiri Abolfazl (red cluster), with strong connections to Ramezanalizadeh Fatemeh, Astaneh Shakiba Darvish Alipour, Sefid Fatemeh, and Khalili Saeed. The blue cluster, represented by Badmasti Farzad, included collaborators such as Ajdary Soheila and Shahcheraghi Fereshteh. Additional collaboration groups were identified, including a purple cluster centered on Doosti Abbas and an orange cluster led by Hashemi Ali. Betweenness centrality analysis (**Figure 5B**) demonstrated that Rasooli Iraj occupied the most prominent bridging position within the author network (65.518), followed by Badmasti Farzad (44.000), Jahangiri Abolfazl (6.177), Rahbar Mohammad Reza (0.746), and Khalili Saeed (0.452).

The country collaboration network (**Figure 5C**) demonstrated a geographically diverse but uneven collaboration structure. The red cluster, dominated by Iran and the United States, exhibited extensive connections with countries including Malaysia, Spain, Canada, France, Lithuania, and Portugal. The blue cluster centered on China showed collaborations with Singapore, Ethiopia, and the Netherlands. In addition, a green cluster was identified around India, involving Egypt, Russia, Saudi Arabia, Pakistan, and South Korea, whereas a purple cluster comprised Australia, Thailand, Japan, Sweden, the United Kingdom, and Germany. Betweenness centrality analysis (**Figure 5D**) showed that the United States had the highest betweenness centrality (107.684), suggesting a prominent bridging role in the international collaboration network. This was followed by China (71.516), Iran (31.584), Canada (28.000), and the United Kingdom (27.754).

The institutional collaboration network (**Figure 5E**) further revealed several major cooperative clusters. The largest cluster was formed around Shahed University and Baqiyatallah University of Medical Sciences (red cluster), with connections to institutions such as Shiraz University of Medical Science and Semnan University. The blue cluster consisted primarily of Islamic Azad University, Tehran University of Medical Sciences, and Pasteur Institute of Iran. Additional clusters included a green cluster involving Case Western Reserve University, University of California, Los Angeles, and University System of Ohio, as well as an orange cluster containing Army Medical University and Sichuan University. Betweenness centrality analysis (**Figure 5F**) indicated that Islamic Azad University had the highest betweenness centrality among institutions (15.071), followed by Baqiyatallah University of Medical Sciences (8.885), University of California, Los Angeles (4.000), Tehran University of Medical Sciences (2.478), and Shahed University (1.948).

### 3.5 Keyword structure and identification of research hotspots

To systematically characterize the knowledge base, thematic evolution, and emerging frontiers of *Acinetobacter baumannii* vaccine research, we performed comprehensive keyword analyses using CiteSpace and the bibliometrix package in R based on the integrated WoSCC–Scopus dataset.

**Figure 6:**
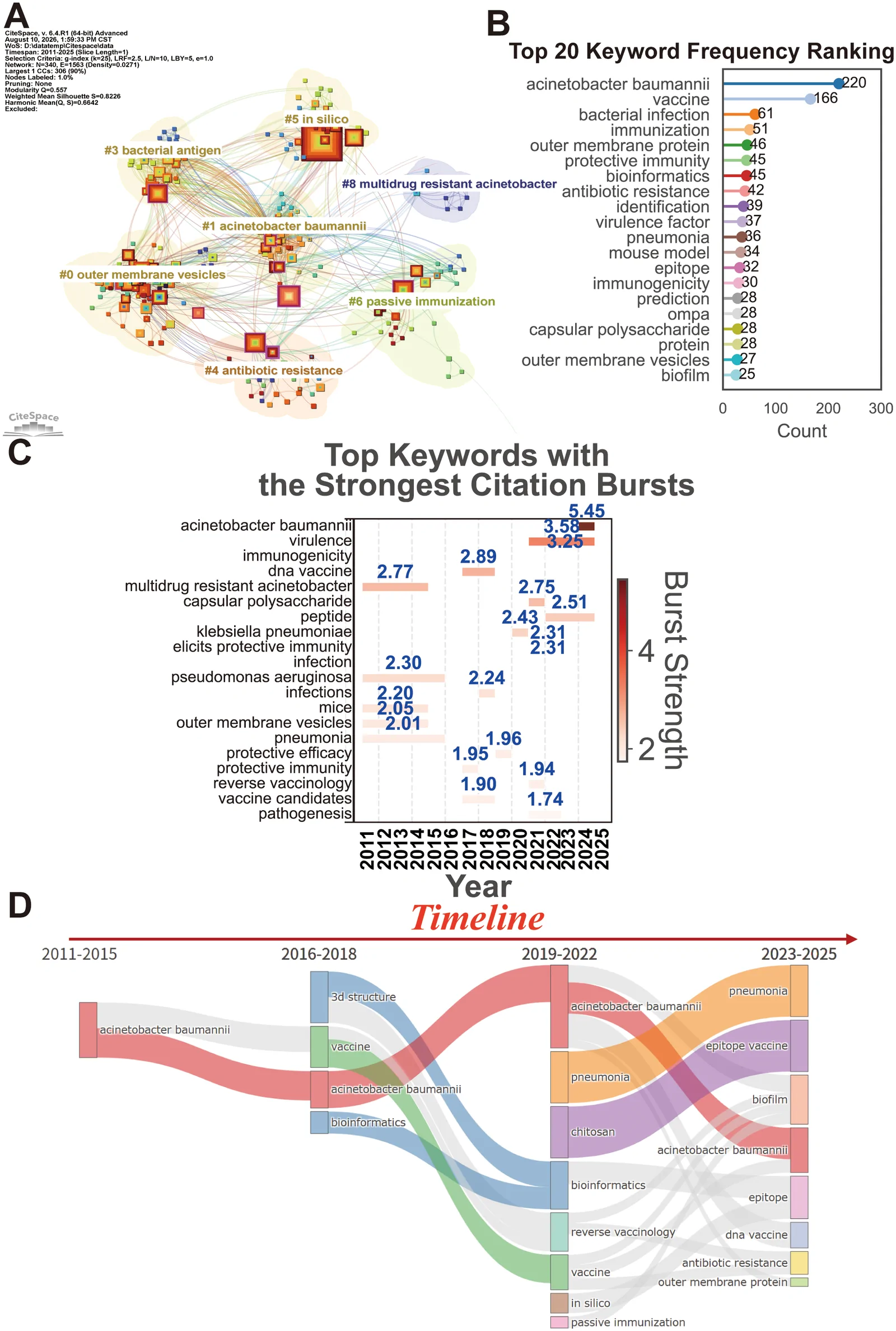
Keyword co-occurrence clustering, high-frequency keyword analysis, keyword burst detection, and Sankey-based thematic evolution. **(A)** CiteSpace keyword co-occurrence network identifying seven clusters (Q = 0.557; S = 0.8226), including outer membrane vesicles, *Acinetobacter baumannii*, bacterial antigens, antibiotic resistance, in silico approaches, passive immunization, and multidrug-resistant Acinetobacter; **(B)** Top 20 high-frequency keywords ranked by occurrence, with *Acinetobacter baumannii* and vaccine representing the most frequently occurring terms; **(C)** Keyword burst detection analysis; **(D)** Sankey diagram depicting thematic evolution across four periods (2011–2015, 2016–2018, 2019–2022, and 2023–2025), showing the progression from early *Acinetobacter baumannii* research toward vaccine development, bioinformatics, reverse vaccinology, epitope-based strategies, and other emerging vaccine-related themes.

Keyword clustering analysis using CiteSpace revealed a well-defined thematic structure within this field. Using the log-likelihood ratio (LLR) method for cluster labeling, the resulting network showed a modularity value of Q = 0.557 and a weighted mean silhouette value of S = 0.8226, suggesting reasonable modularity and high internal consistency of the clusters (**Figure 6A**). Seven major clusters were identified. Cluster #1 (“*Acinetobacter baumannii*”) encompassed studies primarily related to the pathogen itself; clusters #0 (“outer membrane vesicles”) and #3 (“bacterial antigen”) were associated with bacterial structural components and antigen-related research; clusters #4 (“antibiotic resistance”) and #8 (“multidrug resistant acinetobacter”) reflected research on antimicrobial resistance and multidrug-resistant strains; cluster #5 (“in silico”) represented computational approaches, including antigen prediction and immunoinformatics; and cluster #6 (“passive immunization”) was associated with passive immunization strategies. Collectively, these clusters characterize the major thematic domains of research in this field.

Analysis of the top 20 most frequent keywords further highlighted the major research themes (**Figure 6B**). In addition to the retrieval terms “*Acinetobacter baumannii*” (220 occurrences) and “vaccine” (166 occurrences), the most frequently occurring keywords included “bacterial infection” (61 occurrences), “immunization” (51 occurrences), “outer membrane protein” (46 occurrences), “protective immunity” (45 occurrences), “bioinformatics” (45 occurrences), “antibiotic resistance” (42 occurrences), “identification” (39 occurrences), “virulence factor” (37 occurrences), “pneumonia” (36 occurrences), “mouse model” (34 occurrences), “epitope” (32 occurrences), and “immunogenicity” (30 occurrences). These findings indicate that current research encompasses multiple dimensions, including pathogen biology, antigen discovery, immunogenicity assessment, bioinformatic prediction, and animal model validation.

Keyword burst detection further revealed temporal shifts in research attention over time (**Figure 6C**). The keyword “*Acinetobacter baumannii*” exhibited a burst from 2024 to 2025, with the highest burst strength during the study period (5.45). The keyword “virulence” showed a sustained burst from 2021 to 2025, with a burst strength of 3.58, whereas “immunogenicity” exhibited a burst in 2022, with a strength of 3.25. Early-stage burst keywords (approximately 2011–2016) included “multidrug resistant acinetobacter” (2011–2015; strength = 2.77), “*Pseudomonas aeruginosa*” (2011–2016; strength = 2.30), “mice” (2011–2015; strength = 2.20), “outer membrane vesicles” (2011–2015; strength = 2.05), and “pneumonia” (2011–2016; strength = 2.01), indicating early research attention to multidrug resistance, bacterial pathogens and components, respiratory infection, and animal models.

During the intermediate phase (approximately 2017–2020), emerging keywords included “DNA vaccine” (2017–2019; strength = 2.89), “vaccine candidates” (2017–2019; strength = 1.90), “protective immunity” (2017–2018; strength = 1.95), “infections” (2018–2019; strength = 2.24), and “protective efficacy” (2019–2020; strength = 1.96), indicating progressive refinement of vaccine development strategies. In the recent period (after 2020), newly emerging topics included “*Klebsiella pneumoniae*” (2020–2021; strength = 2.43), “elicits protective immunity” (2021; strength = 2.31), “infection” (2021; strength = 2.31), “capsular polysaccharide” (2021–2022; strength = 2.75), “reverse vaccinology” (2021–2022; strength = 1.94), “pathogenesis” (2021–2023; strength = 1.74), and “peptide” (2022–2025; strength = 2.51). These findings suggest that polysaccharide-based antigens, reverse vaccinology approaches, and peptide vaccine design have emerged as important research directions in recent years.

The Sankey diagram further illustrated the temporal evolution of research themes in this field (**Figure 6D**). During 2011–2015, “*Acinetobacter baumannii*” represented the predominant research theme. Between 2016 and 2018, research themes expanded to include “3D structure”, “vaccine”, and “bioinformatics”, suggesting growing interest in computational and structure-based approaches alongside vaccine research. The period from 2019 to 2022 was characterized by substantial thematic diversification, with themes including “pneumonia”, “chitosan”, “bioinformatics”, “reverse vaccinology”, “vaccine”, “in silico”, and “passive immunization”, reflecting broader methodological and immunological research interests. From 2023 to 2025, the thematic landscape further evolved to include “pneumonia”, “epitope vaccine”, “biofilm”, “*Acinetobacter baumannii*”, “epitope”, “DNA vaccine”, “antibiotic resistance”, and “outer membrane protein”. The appearance of “epitope vaccine”, “epitope”, and “DNA vaccine” during this period suggests continued diversification of vaccine-related research themes.

### 3.6 Citation analysis

**Figure 7:**
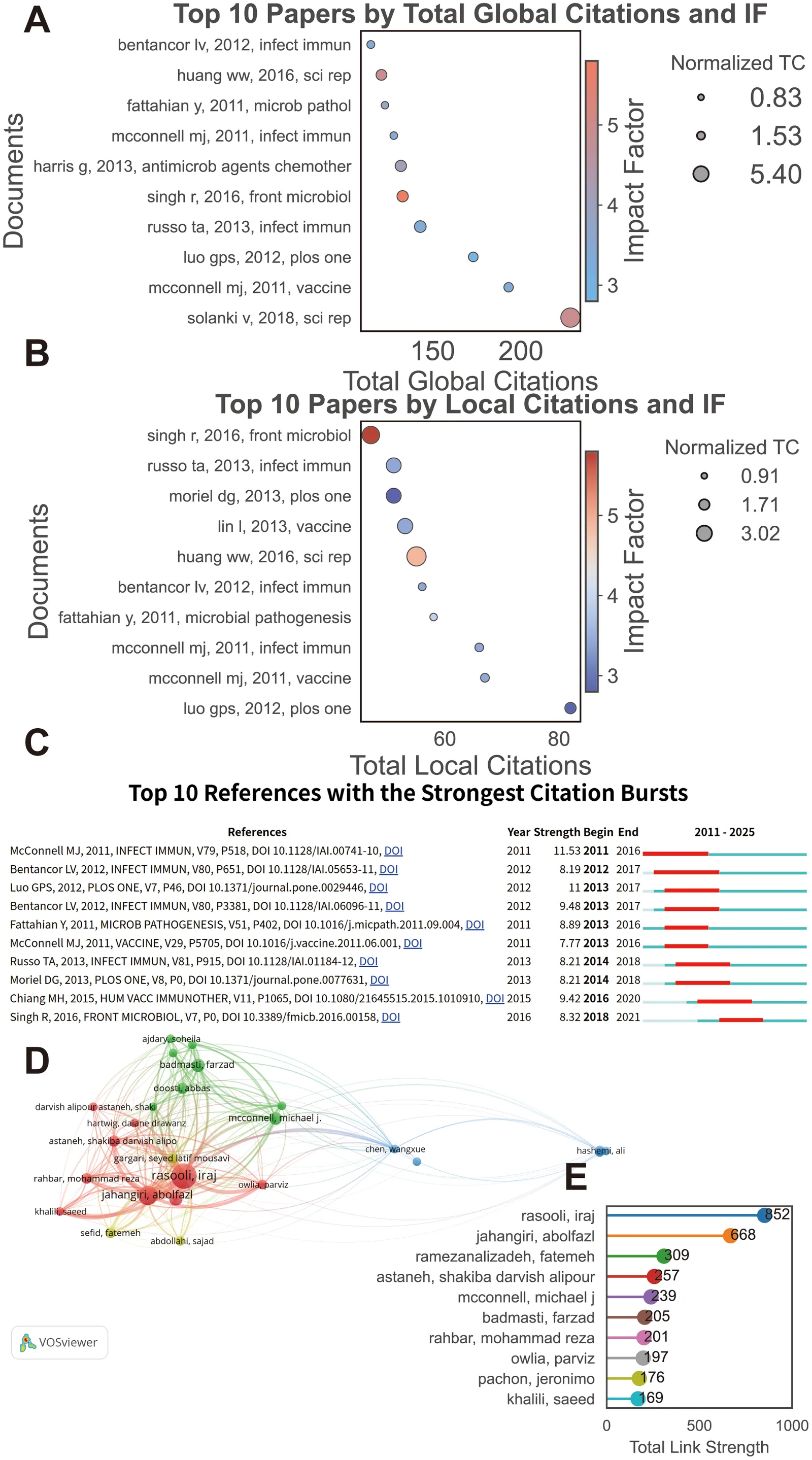
Global and local citation impact, citation bursts, and author co-citation analysis. **(A)** Top 10 documents ranked by total global citations (i.e., the total number of times a document has been cited worldwide), with bubble color indicating the 2025 journal impact factor **(IF)** and bubble size indicating the normalized total global citations. **(B)** Top 10 documents ranked by total local citations (i.e., the number of times a document has been cited within the 243 documents included in this study), with bubble color indicating the 2025 journal impact factor **(IF)** and bubble size indicating the normalized total local citations. **(C)** Top 10 references with the strongest citation bursts. **(D)** Author co-citation network constructed using VOSviewer, illustrating the intellectual connections and co-citation relationships among highly cited authors. **(E)** Top 10 co-cited authors ranked by total link strength (TLS), with Rasooli Iraj ranking highest (852), followed by Jahangiri Abolfazl (668).

This study systematically characterized the citation landscape of *Acinetobacter baumannii* vaccine research through global and local citation impact, citation bursts, and author co-citation analysis, supplemented by a journal dual-map overlay to illustrate citation relationships across journal domains. Based on the integrated WoSCC–Scopus dataset, the top 10 publications ranked by global total citations (Global TC) and local citations (Local Citations) were identified (**Figures 7A, B**; **Tables 5**, **6**). Global citations reflect the overall academic influence of publications across disciplines, whereas local citations represent their contribution and connectivity within the knowledge network of *Acinetobacter baumannii* vaccine research. The combination of these two metrics enables a more comprehensive identification of influential studies.

**Table 5:** Top ten publications ranked by global total citations.

Paper	DOI	Global total citations	Normalized TC	IF
solanki v, 2018, sci rep	10.1038/s41598-018-26689-7	228	5.40	4.9
mcconnell mj, 2011, vaccine	10.1016/j.vaccine.2011.06.001	193	1.30	3.4
luo gps, 2012, plos one	10.1371/journal.pone.0029446	173	1.36	2.8
russo ta, 2013, infect immun	10.1128/IAI.01184-12	143	2.00	3.4
singh r, 2016, front microbiol	10.3389/fmicb.2016.00158	133	1.86	5.8
harris g, 2013, antimicrob agents chemother	10.1128/AAC.00944-13	132	1.85	4.1
mcconnell mj, 2011, infect immun	10.1128/IAI.00741-10	128	0.86	3.4
fattahian y, 2011, microb pathol	10.1016/j.micpath.2011.09.004	123	0.83	3.9
huang ww, 2016, sci rep	10.1038/srep37242	121	1.70	4.9
bentancor lv, 2012, infect immun	10.1128/IAI.06096-11	115	0.91	3.4

**Table 6:** Top ten publications ranked by local citations.

Paper	DOI	Local citations	Normalized local citations	IF
luo gps, 2012, plos one	10.1371/journal.pone.0029446	82	1.39	2.8
mcconnell mj, 2011, vaccine	10.1016/j.vaccine.2011.06.001	67	1.05	3.4
mcconnell mj, 2011, infect immun	10.1128/IAI.00741-10	66	1.04	3.4
fattahian y, 2011, microbial pathogenesis	10.1016/j.micpath.2011.09.004	58	0.91	3.9
bentancor lv, 2012, infect immun	10.1128/IAI.06096-11	56	0.95	3.4
huang ww, 2016, sci rep	10.1038/srep20724	55	3.02	4.9
lin l, 2013, vaccine	10.1016/j.vaccine.2012.11.008	53	2.11	3.4
moriel dg, 2013, plos one	10.1371/journal.pone.0077631	51	2.03	2.8
russo ta, 2013, infect immun	10.1128/IAI.01184-12	51	2.03	3.4
singh r, 2016, front microbiol	10.3389/fmicb.2016.00158	47	2.58	5.8

Comparison of the top 10 Global TC and Local Citations publications revealed substantial overlap between the two rankings, with seven publications appearing in both lists (**Figures 7A, B**; **Tables 5**, **6**). Among these, Luo GPS et al. (2012, *PLOS ONE*; Global TC = 173; Local Citations = 82) received the highest number of local citations. The study identified OmpA as a predominant target of the humoral immune response following *Acinetobacter baumannii* infection and demonstrated that immunization with recombinant OmpA (rOmpA) enhanced survival and reduced tissue bacterial burdens in infected mice (Luo et al., 2012). Other overlapping studies included investigations of OMV-based vaccines by McConnell MJ et al. (2011, *Vaccine*; Global TC = 193; Local Citations = 67) and outer membrane complex (OMC)-based vaccines (2011, *Infection and Immunity*; Global TC = 128; Local Citations = 66) (McConnell et al., 2011b; McConnell et al., 2011a); Bap protein-based vaccine development by Fattahian Y et al. (2011, *Microbial Pathogenesis*; Global TC = 123; Local Citations = 58) (Fattahian et al., 2011); Ata autotransporter-based vaccine evaluation by Bentancor LV et al. (2012, *Infection and Immunity*; Global TC = 115; Local Citations = 56) (Bentancor et al., 2012); K1 capsular polysaccharide-targeted immunotherapy by Russo TA et al. (2013, *Infection and Immunity*; Global TC = 143; Local Citations = 51) (Russo et al., 2013); and the in silico identification of FilF with subsequent *in vivo* validation as a vaccine candidate by Singh R et al. (2016, *Frontiers in Microbiology*; Global TC = 133; Local Citations = 47) (Singh et al., 2016).

Publications appearing exclusively in the Global TC ranking included Solanki V and Tiwari V (2018, *Scientific Reports*; Global TC = 228), Harris G et al. (2013, *Antimicrobial Agents and Chemotherapy*; Global TC = 132), and Huang W et al. (2016, *Scientific Reports*; Global TC = 121). Solanki and Tiwari applied subtractive proteomics combined with reverse vaccinology to identify potential surface antigens for vaccine development (Solanki and Tiwari, 2018), whereas Harris et al. established a highly virulent pneumonia mouse model that has been widely used in experimental infection studies (Harris et al., 2013). Huang et al. evaluated Escherichia coli-derived outer membrane vesicles (OMVs) as an antigen delivery platform against *Acinetobacter baumannii* infection (Huang et al., 2016a).

Conversely, publications identified exclusively through Local Citations included Huang WW et al. (2016, *Scientific Reports*; Local Citations = 55), which identified the 22-kDa outer membrane protein Omp22 and validated its protective efficacy (Huang et al., 2016b); Lin L et al. (2013, *Vaccine*; Local Citations = 53), which investigated the effects of rOmpA vaccine dosage and immune epitopes (Lin et al., 2013); and Moriel DG et al. (2013, *PLOS ONE*; Local Citations = 51), which combined reverse vaccinology and proteomics to identify candidate vaccine antigens (Moriel et al., 2013).

The substantial overlap between the two citation rankings suggests that studies focusing on prominent vaccine targets, such as OmpA, Bap, Ata, K1 capsular polysaccharide, and FilF, together with vaccine strategies involving OMVs and outer membrane complexes (OMCs), constitute a core component of the knowledge base of *Acinetobacter baumannii* vaccine research. Meanwhile, differences between Global TC and Local Citations highlight their complementary roles, with the former reflecting broader interdisciplinary influence and the latter capturing field-specific scientific contributions.

Citation burst detection identified several influential publications with strong temporal impact (**Figure 7C**). The studies by McConnell MJ et al. (2011) and Luo GPS et al. (2012) exhibited the strongest citation bursts, with strengths of 11.53 and 11.00, respectively. The burst for McConnell MJ et al. began in 2011 and persisted through 2016, whereas that for Luo GPS et al. extended from 2013 to 2017, highlighting the sustained attention received by these early studies. In contrast, Singh R et al. (2016) displayed the most recent citation burst among the top-ranked references (strength = 8.32; 2018–2021), suggesting continued interest in emerging vaccine candidates during the later development of the field.

Co-cited author analysis using VOSviewer revealed a densely interconnected network comprising several author clusters (**Figure 7D**). Rasooli Iraj and Jahangiri Abolfazl occupied particularly prominent positions and showed extensive co-citation relationships with other authors in the network. Consistently, total link strength analysis demonstrated that Rasooli Iraj had the highest co-citation connectivity (TLS = 852), followed by Jahangiri Abolfazl (TLS = 668) and Ramezanalizadeh Fatemeh (TLS = 309) (**Figure 7E**). These findings highlight their prominent positions within the co-citation structure of *Acinetobacter baumannii* vaccine research.

As a complementary analysis, the journal dual-map overlay showed the major citation trajectories between citing and cited journal domains (**Supplementary Figure 1**), providing an overview of the disciplinary structure underlying the citation network.

## 4 Discussion

### 4.1 Evolution of *Acinetobacter baumannii* vaccine development from whole-cell to defined antigen-based platforms

The development of vaccines against *Acinetobacter baumannii* has progressively evolved from broad antigenic preparations toward increasingly defined and rationally designed vaccine platforms. Early studies using inactivated whole-cell vaccines demonstrated that protective immunity against *Acinetobacter baumannii* could be achieved, providing important proof-of-concept for vaccination against this pathogen and establishing an experimental foundation for the subsequent development of acellular and antigen-defined strategies (McConnell and Pachon, 2010; Shu et al., 2016). Although the study by McConnell and Pachón was published in 2010 and therefore falls outside the predefined 2011–2025 bibliometric window, it is cited here as historical context for the early development of *Acinetobacter baumannii* vaccination and was not included in the quantitative bibliometric analysis.

Whole-cell formulations offer the potential advantage of presenting a broad repertoire of bacterial antigens and may therefore induce immune responses against multiple targets simultaneously. However, their complex antigenic composition, together with the marked phenotypic and antigenic heterogeneity among *Acinetobacter baumannii* strains, may complicate vaccine standardization and limit consistent cross-strain protection (Garcia-Quintanilla et al., 2013; Valcek et al., 2023). These considerations have encouraged the development of acellular and defined-antigen approaches, which allow more precise control over vaccine composition and facilitate the rational selection and evaluation of protective antigens.

The citation landscape identified in the present study reflects an important stage in this evolution. Seminal studies by McConnell et al. demonstrated protective immunity induced by OMC- and OMV-based vaccines, whereas Luo et al. established OmpA as an important protective antigen (McConnell et al., 2011a; Luo et al., 2012). These publications showed high local citation impact and prominent citation bursts, underscoring their sustained influence on subsequent *Acinetobacter baumannii* vaccine research. Subsequent investigations further expanded the repertoire of defined vaccine targets. Omp22 and FilF, for example, showed protective potential in experimental models, with FilF providing an early example of a candidate identified computationally and subsequently validated *in vivo (*Huang et al., 2016b; Singh et al., 2016). Other surface-exposed or virulence-associated components, including Bap, Ata, and capsular polysaccharides, have also been investigated as potential immunological targets, further broadening the antigenic landscape available for vaccine development (Garcia-Quintanilla et al., 2013).

The bibliometric findings further support this progression. The prominence of “outer membrane protein”, together with “bioinformatics”, “epitope”, and “immunogenicity” in the keyword landscape, reflects the coexistence of conventional antigen-focused research and increasingly rational approaches to vaccine design. Together with the citation structure of the field, these findings suggest progressive diversification rather than simple replacement of earlier vaccine platforms. This evolving landscape provides the basis for the development of increasingly defined, multicomponent, and rationally designed vaccine strategies (Garcia-Quintanilla et al., 2013; Ud-Din et al., 2022).

### 4.2 Outer membrane vesicles and mucosal immunization strategies

OMVs provide a naturally derived multivalent platform in which multiple outer membrane antigens are presented within their native membrane environment, offering a potential advantage over vaccines based on a single recombinant antigen (McConnell et al., 2011b; Weng et al., 2023). Early experimental work demonstrated that *Acinetobacter baumannii* OMV immunization elicited antibody responses against multiple bacterial antigens and protected mice against both reference and clinically resistant strains (McConnell et al., 2011b). In parallel, our bibliometric analysis identified “outer membrane vesicles” as a distinct thematic cluster and an early burst keyword, while the seminal OMV/OMC studies occupied prominent positions in the citation landscape. Together, these findings indicate that OMV-based vaccination represents both an experimentally established platform and an important component of the intellectual foundation of *Acinetobacter baumannii* vaccine research (McConnell et al., 2011b; McConnell et al., 2011a; Weng et al., 2023).

Subsequent studies have focused on improving OMV formulation, consistency, and safety. Importantly, different preparation procedures can substantially alter OMV yield, particle size, protein composition, and lipopolysaccharide (LPS) content, with corresponding differences in immunogenicity and protective efficacy (Li et al., 2020). Nanoparticle-supported delivery represents one strategy to improve formulation performance; an OMV-coated nanoparticle vaccine induced functional antibody responses and protected mice against both pneumonia and sepsis (Bjanes et al., 2023). Other engineering approaches have directly addressed endotoxin-associated concerns. LPS-deficient *Acinetobacter baumannii* was shown to produce OMVs that retained immunogenicity and protective activity despite the absence of LPS (Pulido et al., 2020), while lysin-generated OMVs exhibited improved homogeneity and reduced endotoxin content and cytotoxicity while maintaining protection in experimental infection models (Li et al., 2024). Collectively, these studies suggest that OMV engineering may improve platform reproducibility and safety without necessarily sacrificing protective immunogenicity (Li et al., 2020; Pulido et al., 2020; Bjanes et al., 2023; Li et al., 2024).

The route of immunization represents another important determinant of vaccine-induced protection, particularly against respiratory *Acinetobacter baumannii* infection. Direct comparison of intranasal and intramuscular administration has shown that intranasal OMV vaccination induces antigen-specific mucosal secretory immunoglobulin A (sIgA) in addition to systemic antibody responses and can provide stronger protection than intramuscular vaccination in experimental models (Li et al., 2020). Consistent with this observation, Higham et al. found that intranasal immunization with OMVs derived from clinically relevant *Acinetobacter baumannii* isolates induced systemic IgG and IgM together with respiratory IgA, markedly reduced airway bacterial burden, and prevented systemic dissemination, whereas parenteral immunization provided less complete protection against respiratory challenge (Higham et al., 2023). The relevance of mucosal delivery is not restricted to OMVs: intranasal immunization with recombinant OmpA also elicited both systemic and mucosal antibody responses and improved survival following challenge with multidrug-resistant isolates (Zhang et al., 2016), while a recent intranasal chitosan–poly(lactic-co-glycolic acid) (PLGA)–rOmp22 nanovaccine generated sustained local and systemic immunity and protected mice against *Acinetobacter baumannii*-associated pneumonia (Yang et al., 2025). Taken together, these independent studies support the concept that establishing immunity at the respiratory mucosa may complement systemic responses and improve protection against airway colonization and pulmonary infection (Zhang et al., 2016; Li et al., 2020; Higham et al., 2023; Yang et al., 2025).

Despite these encouraging findings, several barriers continue to limit the translation of OMV-based vaccines. OMV composition is sensitive to bacterial strain and production method, and experimentally prepared *Acinetobacter baumannii* OMVs differ in particle size, protein cargo, and LPS content (Li et al., 2020). In particular, LPS represents a major safety consideration because its intrinsic immunostimulatory activity may also contribute to excessive reactogenicity at vaccine-relevant doses (Pulido et al., 2020; Weng et al., 2023). Proof-of-concept studies using LPS-deficient OMVs and alternative vesicle-generation methods demonstrate that endotoxin burden can be substantially reduced while retaining protective immunogenicity (Pulido et al., 2020; Li et al., 2024). However, these strategies have so far been evaluated predominantly in preclinical models, and questions regarding manufacturing reproducibility, antigenic consistency across strains, durability of protection, and optimal route and dose remain unresolved (Li et al., 2020; Pulido et al., 2020; Bjanes et al., 2023; Li et al., 2024).

Thus, the major challenge for OMV-based vaccination is no longer simply to demonstrate immunogenicity, but to translate a biologically attractive multivalent platform into a standardized, safe, and broadly protective vaccine product.

### 4.3 Targeting virulence determinants and bacterial survival mechanisms

The keyword landscape indicates increasing interest in the pathogenic functions underlying *Acinetobacter baumannii* infection. “Virulence factor” was among the frequently occurring keywords, while “virulence” showed a sustained burst from 2021 to 2025 and “pathogenesis” emerged during 2021–2023; “biofilm” also appeared in the most recent phase of thematic evolution. Rather than representing a shift away from antigen discovery, these patterns suggest that vaccine target selection is increasingly being considered in the context of bacterial functions that contribute to colonization, persistence, and disease progression.

Biofilm formation is particularly relevant to this function-oriented strategy because it contributes to surface persistence, host colonization, and tolerance to environmental and antimicrobial stresses (Mea et al., 2021). Several vaccine candidates are themselves closely linked to these pathogenic processes. Bap contributes to intercellular adhesion and mature biofilm formation, and immunization with a recombinant Bap fragment provided early evidence that a biofilm-associated virulence determinant could also serve as a protective antigen (Fattahian et al., 2011). OmpA similarly participates in adhesion, biofilm formation, and host–pathogen interactions, supporting its dual relevance as both a pathogenic determinant and an immunological target (Uppalapati et al., 2020). More recently, combined immunization with Bap and Oma87 produced enhanced protection in a murine sepsis model, further supporting the potential value of incorporating functionally relevant surface antigens into vaccine design (Mansouri et al., 2023).

The value of linking antigen selection to bacterial fitness and virulence is also supported by studies of other functionally important surface proteins. Peptidoglycan-associated lipoprotein (PAL), for example, contributes to bacterial fitness and virulence, and deletion of PAL attenuated disease in a murine pneumonia model; immunization with recombinant PAL subsequently provided partial protection against *Acinetobacter baumannii* challenge (Zeng et al., 2023). Similarly, a quadrivalent vaccine combining antigens associated with planktonic growth and biofilm formation—BauA, HemTR, CsuA/B, and FimA—elicited antibody responses and protected mice against carbapenem-resistant clinical isolates (Ramezanalizadeh et al., 2022). These findings suggest that considering antigen expression and function across different bacterial physiological states may provide a biologically informed strategy for vaccine target selection.

Nevertheless, functional involvement in virulence does not by itself establish an antigen as an optimal vaccine target. Antigen expression, surface accessibility, conservation across circulating strains, and the capacity to induce reproducible protective immunity are equally important considerations (Mea et al., 2021; Valcek et al., 2023). The marked phenotypic heterogeneity of contemporary *Acinetobacter baumannii* isolates therefore argues against prioritizing candidate antigens solely on the basis of a single pathogenic function. Instead, integrating biological relevance with antigen conservation and experimental protection may provide a more robust framework for target selection.

### 4.4 Conserved antigens and multi-epitope vaccine design

Antigenic and phenotypic heterogeneity among *Acinetobacter baumannii* strains remains a major obstacle to broad vaccine protection (Valcek et al., 2023). This has encouraged the prioritization of conserved and immunogenic antigens. Omp22, for example, shows a high degree of sequence conservation and has demonstrated protection in both active- and passive-immunization models (Huang et al., 2016b). Pangenome and immunoproteomic analyses have further identified conserved surface-associated proteins as potential vaccine targets, providing a systematic basis for improving cross-strain antigen selection (Hassan et al., 2016).

Keyword analysis revealed that “outer membrane protein”, “epitope”, and “immunogenicity” were prominent keywords, while “peptide” showed sustained recent attention. These patterns indicate increasing interest in moving beyond whole-protein antigen selection toward defined protective regions within candidate antigens. Accordingly, computational studies have combined conserved-antigen screening with B-cell and T-cell epitope prediction to construct multi-epitope vaccine candidates (Touhidinia et al., 2021; Ud-Din et al., 2022; Dey et al., 2023). Importantly, this strategy is beginning to extend beyond purely in silico design; an immunoinformatics-guided multi-peptide vaccine has demonstrated protective efficacy and reduced bacterial burdens in a murine pulmonary infection model (Jeffreys et al., 2024).

Nevertheless, multi-epitope design does not by itself ensure broad or durable protection. Candidate epitopes must be conserved, accessible and expressed during infection, and retain immunogenicity when incorporated into multicomponent constructs (Hassan et al., 2016; Dey et al., 2023; Jeffreys et al., 2024). Moreover, many reported candidates remain supported mainly by computational evaluation rather than direct challenge experiments (Ud-Din et al., 2022; Beig et al., 2025). Thus, translating computationally prioritized epitopes into experimentally validated antigen combinations with reproducible cross-strain efficacy remains a key challenge.

### 4.5 Reverse vaccinology, AI-assisted design, and emerging mRNA platforms

The bibliometric findings indicate an increasing integration of computational approaches into *Acinetobacter baumannii* vaccine research. “Bioinformatics” was among the prominent keywords, “in silico” formed a distinct thematic cluster, and reverse vaccinology emerged during the recent phase of thematic evolution. These patterns are consistent with studies using genome-based screening and immunoinformatics to prioritize conserved, surface-exposed, and potentially immunogenic antigens before experimental evaluation (Chiang et al., 2015; Ahmad and Azam, 2018; Shahid et al., 2019; Fereshteh et al., 2020).

Reverse vaccinology has broadened antigen discovery beyond candidates identified by conventional experimental screening, while epitope prediction, structural modeling, and related computational tools have facilitated more systematic prioritization of vaccine targets (Chiang et al., 2015; Ahmad and Azam, 2018; Fereshteh et al., 2020). Machine learning approaches may further extend these pipelines by integrating multiple antigen features and improving candidate ranking, although their role in *Acinetobacter baumannii* vaccine development remains emerging rather than established (Nasir et al., 2024). Thus, computational methods are best viewed as tools for narrowing the antigen search space rather than substitutes for experimental validation.

Recent mRNA studies provide an important extension of this computationally guided antigen-selection strategy. Immunoinformatics-based studies have incorporated conserved antigens and predicted B- and T-cell epitopes into multi-epitope mRNA constructs, although these candidates have so far been evaluated primarily in silico (Xu et al., 2024; Ma et al., 2025). More importantly, experimental studies have now provided direct proof-of-concept. Liu et al. developed mRNA-lipid nanoparticle (LNP) vaccines encoding SmpA-PLD and OmpK-Omp22, both of which induced humoral and cellular responses and protected mice against *Acinetobacter baumannii*, with OmpK-Omp22 showing greater protection in a sepsis model (Liu et al., 2025). Higham et al. independently identified OXA-23 and PAL as conserved antigens and showed that mRNA vaccination reduced bacterial burden after carbapenem-resistant *Acinetobacter baumannii* challenge; intranasal delivery of OXA-23 mRNA further enhanced airway bacterial clearance (Higham et al., 2025).

These findings position mRNA as a flexible platform for translating computationally prioritized protein antigens into multivalent vaccine formulations, while also linking antigen selection with alternative delivery routes. However, mRNA should be regarded as an enabling platform rather than a universal solution for antibacterial vaccination. Its translational value will depend on appropriate antigen selection, delivery, durability of protection, and demonstration of reproducible efficacy across clinically diverse strains (Davido et al., 2026). Accordingly, the next step is not simply to expand the number of computationally predicted or mRNA-encoded candidates, but to integrate antigen discovery with rigorous *in vivo* validation and clinically relevant infection models.

**Figure 8:**
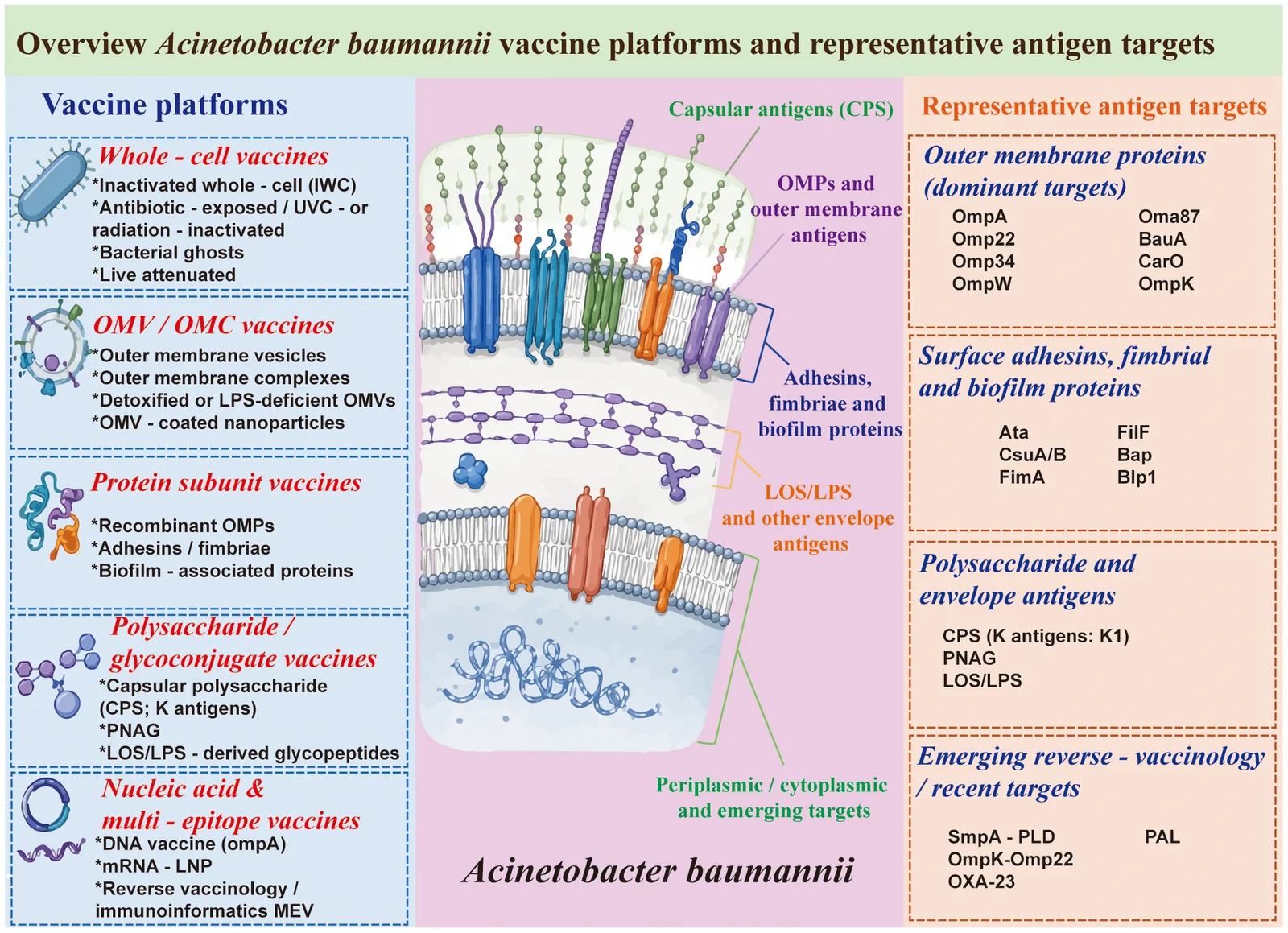
Overview of *Acinetobacter baumannii* vaccine platforms and representative antigen targets. The left panel summarizes five major vaccine platforms, including whole-cell vaccines, outer membrane vesicle/outer membrane complex (OMV/OMC) vaccines, protein subunit vaccines, polysaccharide/glycoconjugate vaccines, and nucleic acid or multi-epitope vaccines. The central panel illustrates the major structural compartments of *Acinetobacter baumannii* and the localization of candidate antigens, including capsular, outer membrane, adhesin/fimbrial and biofilm-associated, LOS/LPS-associated, and periplasmic/cytoplasmic targets. The right panel classifies representative antigens into outer membrane proteins, surface adhesins/fimbrial and biofilm-associated proteins, polysaccharide and envelope antigens, and emerging targets identified through reverse vaccinology and related computational approaches.

Collectively, these developments illustrate the increasing diversification of *Acinetobacter baumannii* vaccine development, encompassing whole-cell, OMVs, defined subunit, multi-epitope, DNA, and emerging mRNA-based approaches directed against a broad range of structural and virulence-associated antigens. The principal vaccine platforms and representative antigen targets discussed above are summarized in **Figure 8**.

### 4.6 Clinical translation challenges and future perspectives

Despite the continued expansion of *Acinetobacter baumannii* vaccine research, clinical translation remains limited. Our analysis showed an annual publication growth rate of 18.68%, although research output varied substantially across years. This growing scientific interest is consistent with the urgent need for preventive strategies against carbapenem-resistant *Acinetobacter baumannii*, which contributes substantially to the global burden of antimicrobial resistance (Antimicrobial Resistance C., 2022). Nevertheless, no licensed *Acinetobacter baumannii* vaccine is currently available, and previous reviews have identified candidate selection, formulation, and inconsistent preclinical validation as persistent barriers to advancement (Ma and Chen, 2021; Lau and Tan, 2024). Thus, increasing research activity has not yet translated into comparable progress toward clinical application.

A major challenge concerns the target population: *Acinetobacter baumannii* infections disproportionately affect critically ill patients, particularly those in intensive care units, individuals with compromised immunity, and patients requiring prolonged mechanical ventilation (Garnacho-Montero et al., 2015). These populations may respond suboptimally to active immunization, while vaccination after hospitalization may provide insufficient time to establish protective immunity (Ma and Chen, 2021). Consequently, defining appropriate target groups and the optimal timing of vaccination remains an important consideration for future clinical development.

Antigenic diversity represents a second major barrier to broad protection. Contemporary clinical isolates exhibit substantial phenotypic heterogeneity, while surface structures such as capsular polysaccharides can vary markedly among strains (Singh et al., 2018; Valcek et al., 2023). Such variability may limit the generalizability of single-antigen vaccines and reinforces the need to prioritize conserved, accessible antigens and to evaluate candidate vaccines against genetically and geographically diverse clinical isolates (Hassan et al., 2016). Multi-antigen and multicomponent approaches may provide broader coverage, but their cross-strain efficacy requires direct experimental confirmation.

A further limitation is the gap between preclinical models and human infection. “Mouse model” was among the prominent terms identified in the keyword analysis, reflecting the central role of murine systems in evaluating *Acinetobacter baumannii* vaccine efficacy. Although established pneumonia models provide valuable tools for comparing candidate vaccines (Bergamini et al., 2021), differences in challenge strains, infection routes, vaccine doses, adjuvants, and outcome measures can complicate comparisons across studies. Recent reviews have similarly highlighted inconsistencies among validation protocols as a major obstacle to candidate prioritization (Lau and Tan, 2024). Greater standardization of preclinical models, together with assessment of immune durability and protection across multiple clinically relevant strains, will therefore be essential for reliable down-selection of vaccine candidates.

Future progress will depend less on expanding the number of vaccine candidates than on establishing an integrated framework for their comparative evaluation and translational prioritization (Ma and Chen, 2021; Lau and Tan, 2024). Computational antigen discovery and emerging platforms, including multi-epitope and mRNA vaccines, can accelerate candidate generation (Ud-Din et al., 2022; Higham et al., 2025; Liu et al., 2025), but their translational value will ultimately depend on rigorous *in vivo* validation, standardized manufacturing, and clinically relevant efficacy criteria (Ma and Chen, 2021; Lau and Tan, 2024). The relatively modest international collaboration rate observed in this study (19.75%) also highlights an opportunity for broader multinational studies incorporating geographically diverse clinical isolates and harmonized experimental protocols. Such efforts may facilitate the transition from an increasingly diverse preclinical vaccine portfolio toward a smaller number of well-characterized candidates with reproducible cross-strain efficacy and realistic potential for clinical development.

Overall, advancing *Acinetobacter baumannii* vaccines toward clinical application will require greater emphasis on candidate prioritization, standardized validation, and cross-strain evaluation rather than continued expansion of the preclinical candidate pool.

### 4.7 Limitations

This study has several limitations that should be acknowledged when interpreting the findings. First, the analysis was restricted to English-language articles and reviews indexed in the Web of Science Core Collection and Scopus. Consequently, relevant studies published in other languages may have been omitted, potentially leading to underrepresentation of research from certain countries or regions and affecting the observed geographic, collaborative, or thematic patterns. The magnitude and direction of this potential bias cannot be determined from the present dataset. Future bibliometric studies incorporating multilingual sources or targeted retrieval of non-English literature may provide a more comprehensive representation of the global research landscape.

Second, although data were retrieved from both the Web of Science Core Collection and Scopus, differences in coverage, indexing policies, update frequency, and document types may introduce systematic bias. We attempted to minimize this through data integration and normalization, but variations in database structure and indexing rules may still influence institutional attribution and co-authorship network accuracy.

Third, limitations in automated author and affiliation disambiguation may result in residual errors. Despite standardized cleaning procedures, inconsistencies in naming conventions and institutional reporting may lead to minor misclassification, particularly for authors with similar names or variable affiliations.

Finally, bibliometric analysis is a retrospective, macro-level approach used to describe research output, knowledge structure, and thematic evolution. It is not designed to infer mechanisms or establish causality. Therefore, the results should be interpreted as a quantitative overview of the development of *Acinetobacter baumannii* vaccine research rather than evidence of biological or clinical causation.

## 5 Conclusion

This bibliometric analysis maps the development of *Acinetobacter baumannii* vaccine research from 2011 to 2025, revealing substantial growth, concentrated geographic contributions, and progressive diversification of vaccine platforms and antigen-selection strategies. OMV/OMC-based vaccines and defined antigens remain central to the field, while bioinformatics, reverse vaccinology, peptide/epitope-based design, and emerging mRNA approaches are expanding the scope of rational vaccine development.

However, antigenic heterogeneity, limited cross-strain validation, and variability in preclinical evaluation continue to hinder translation. Future efforts should prioritize conserved and functionally relevant targets, standardized and clinically relevant validation frameworks, and broader international collaboration to facilitate rational candidate selection and advancement toward clinical application.

## Data Availability

The original contributions presented in the study are included in the article/**Supplementary Material**. Further inquiries can be directed to the corresponding author.
